# Comparison of Paeoniflorin and Albiflorin on Human CYP3A4 and CYP2D6

**DOI:** 10.1155/2015/470219

**Published:** 2015-05-19

**Authors:** Li-Na Gao, Ye Zhang, Yuan-Lu Cui, Olunga Mary Akinyi

**Affiliations:** ^1^Tianjin State Key Laboratory of Modern Chinese Medicine, Tianjin University of Traditional Chinese Medicine, Tianjin 300193, China; ^2^Research Center of Traditional Chinese Medicine, Tianjin University of Traditional Chinese Medicine, Tianjin 300193, China; ^3^Department of Pharmaceutical Sciences, Zibo Vocational Institute, Zibo, Shandong 255314, China

## Abstract

Peony (*Paeonia lactiflora* Pall-) is a plant medicine and a functional food ingredient with wide application for more than 2000 years. It can be coadministrated with many other drugs, composed of traditional Chinese medicine compound such as shaoyao-gancao decoction. In order to explore the efficacy and safety of peony, effects of paeoniflorin and albiflorin (the principal components of peony) on cytochrome P450 (CYP) 3A4 and CYP2D6 were analyzed in human hepatoma HepG2 cells and evaluated from the level of recombinant CYP enzymes *in vitro*. The findings indicated that albiflorin possessed stronger regulation on the mRNA expression of CYP3A4 and CYP2D6 than paeoniflorin. For the protein level of CYP3A4, albiflorin showed significant induction or inhibition with the concentration increasing from 10^−7^ M to 10^−5^ M, but no remarkable variation was observed in paeoniflorin-treated group. Enzyme activity assay implied that both paeoniflorin and albiflorin could regulate CYP3A4 and CYP2D6 with varying degrees. The results showed that albiflorin should be given more attention because it may play a vital role on the overall efficacy of peony. The whole behavior of both paeoniflorin and albiflorin should be focused on ensuring the rationality and effectiveness of clinical application.

## 1. Introduction

The cytochrome P450 (CYP) enzymes, acting as important metabolic enzymes in humans, are involved in a diverse array of physiological and xenobiotic metabolic pathways. Modulation of CYP450 isoenzymes via inhibition or induction may evoke severe adverse effects such as decreasing the bioavailability of drugs and inducing toxic reaction [1, 2]. The major isoenzymes of human CYP450 isoenzymes mainly consist of CYP3A4, CYP2D6, CYP1A2, CYP2C9, and CYP2C19, and they metabolize approximately 90% of the known drugs* in vivo *[3]. Among them, CYP3A4 catalyzes about 34% of drug oxidation, and CYP2D6-mediated catalysis accounts for about 19%. Inhibition of one of them may result in unexpected clinical drug interactions, especially when coadministrated drugs are metabolized by the same enzyme [4].

With the rapid development of medicine, multiple drug therapy has become common practice in the treatment of diseases. Drug combination is associated with the alteration of CYP450 metabolism, including inhibition (resulting in treatment failures) and induction (leading to toxicity) [5, 6]. Thus, the investigation of herbs or drugs on CYP450 isoenzymes is critical for guiding drug applications. It could provide bases for researching the lead compounds and guiding the use of combination drug therapy in clinic as well.

**Figure 1:**
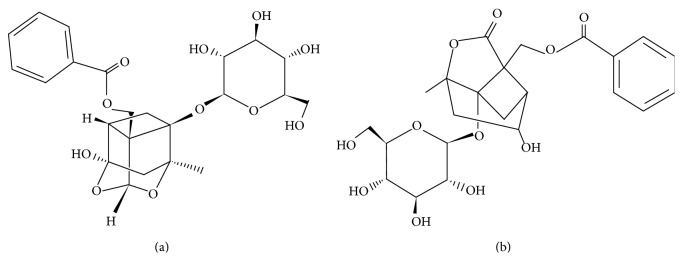
Structures of paeoniflorin and albiflorin. (a) Paeoniflorin. (b) Albiflorin.

*Paeonia lactiflora *Pall., first described in the Chinese materia medica Shen Nong Ben Cao Jing (the pharmacopoeia of Shen Nong, Anonymous, ~200 BC), has been widely used. Paeoniae Radix Alba (Bai-shao) is the steamed and dried root of cultivated* Paeonia lactiflora *Pall., and Paeoniae Radix Rubra (Chi-shao) is the dried root of wild* Paeonia lactiflora *Pall. or* Paeonia veitchii* Lynch. A shorthand name, peony, was given to* Paeonia lactiflora *Pall. in the present study. Peony is awarded the fame of the melody of love and romantic folk songs in ancient China. Being an ornamental plant, the root of peony is used for antidepression [7, 8], antioxidant [9], anticoagulation [10], anti-inflammation [11], liver protection [12], and so on. Moreover, it exerts a purgative action via the direct effects to the intestines. The existing evidences have displayed that the total glucosides of peony could attenuate the expression of CYP2E1 [13]. Paeoniflorin and albiflorin (Figure 1) are extracted from the root of peony which is a famous-region drug in Zhejiang province. Many preparations composed of peony contain albiflorin and paeoniflorin as main the effective constituents [14] and their concentrations are 1.03% and 2.42%, respectively [15]. Paeoniflorin, the principal component of the total glucosides of peony, has been proved to be the inhibitor of CYP1A, and it could be coadministrated with the first-pass effective compounds to enhance the bioavailability of drugs [16]. Albiflorin, another main effective compound, is the isomer of paeoniflorin. However, little focus has been shown on studies of albiflorin [17–19]. Gan et al. [20] have proved that both albiflorin and paeoniflorin have pharmacokinetic interactions with other ingredients of shaoyao-gancao decoction and reduce their systematic exposure level. As shaoyao-gancao decoction, peony can be coadministrated with many other drugs, composed of traditional Chinese medicine compound [21]. It is of great importance to explore the effects of paeoniflorin and albiflorin on CYP450 isoenzymes guiding clinical rational use.

In this study, paeoniflorin and albiflorin on CYP450 isoenzymes were evaluated by a feasible and time-saving complex method, composed of real-time reverse transcription polymerase chain reaction (real-time RT-PCR) and luminogenic CYP assay, and verified by Western blot.

## 2. Materials and Methods

### 2.1. Reagents

Dulbecco's modified Eagle's medium (DMEM, high glucose) and 3-(4, 5-dimethylthiazol-2-yl)-2, 5-diphenyltetrazolium bromide (MTT) were obtained from Sigma-Aldrich Co. (St. Louis, USA). UNIQ-10 column Trizol total RNA extraction kit, anti-GAPDH, and anti-CYP3A4 antibody were obtained from Sangon Biological Engineering Technology & Services Co., Ltd. (Shanghai, China). Anti-CYP2D6 and HRP-conjugated goat anti-rabbit IgG antibody were bought from Abgent (San Diego, USA). FastStart Universal SYBR Green Master (ROX) kit was purchased from Roche (Mannheim, Germany). P450-Glo Assay kits were purchased from Promega (Madison, WI, USA).

### 2.2. Drug Dilutions

Paeoniflorin (purity: 95.2%) and albiflorin (purity: 96.5%) were purchased from Nanjing Zelang Medical Technology Co., Ltd., and Shanghai Forever Biotech Co., Ltd., respectively. Stock solutions of 10^−3^ M were made by dissolving them in phosphate buffer solution. For experiments, they were diluted in DMEM (without phenol red) supplemented with 5% fetal bovine serum (FBS).

### 2.3. Cells and Cell Culture Conditions

The human hepatoma cell line, HepG2, was obtained from Cell Culture Center of Chinese Academy of Medical Sciences (Beijing, China). Cells were maintained at 37°C in 5% CO_2_ in DMEM supplemented with 15% FBS, 0.1% sodium pyruvate, 3.7 g/L sodium bicarbonate, 100 U/mL penicillin, and 100 *μ*g/mL streptomycin. For all experiments, cells were grown to a confluence of 80–90%.

### 2.4. MTT Assay

HepG2 cells, distributed into 96-well plates (1.0 × 10^4^ cells per well) for 24 h, were treated with various concentrations (10^−3^ to 10^−10^ M) of paeoniflorin or albiflorin. 18 h later, MTT was added to a final concentration of 0.5 mg/mL and incubated for additional 2 h at 37°C and 5% CO_2_. After discarding the medium, the formazan precipitate was solubilized in 100 *μ*L DMSO. Then, absorbance was recorded at 570 nm using a multifunctional microplate reader (FlexStation 3, Molecular Devices, USA).

### 2.5. Real-Time RT-PCR

HepG2 cells (5.0 × 10^5^ cells per well) were plated in 6-well plates for 24 h and treated with various concentrations of paeoniflorin or albiflorin (10^−5^ to 10^−7^ M) for additional 24 h. Total RNA was isolated using a Sangon UNIQ-10 column Trizol total RNA extraction kit according to the manufacturer's instructions. Reverse transcriptions were performed using an ImProm-II Reverse Transcription System kit. The reaction volume of 20 *μ*L contains 0.5 *μ*g total RNA. The real-time RT-PCR oligonucleotide primers used for CYP3A4, CYP2D6, and GAPDH (used as an internal control) were as shown in Table 1. The total reaction volume of 25 *μ*L contains 0.5 *μ*L of each forward and reverse primer (0.3 *μ*M final concentrations), 12.5 *μ*L of FastStart Universal SYBR Green Master (ROX), and 2 *μ*L of cDNA. PCR cycles were 95°C for 10 min, 40 cycles of 95°C for 15 s, and 60°C for 1 min. Melting curve analysis was carried out to verify PCR product specificity. The amplification and analysis using relative C_T_ method were conducted in ABI Prism 7500 Real-Time PCR System. The fold increase or decrease depends on a blank control after eliminating a housekeeping gene according to 2^−ΔΔC_T_^ [22, 23].

### 2.6. Western Blotting

HepG2 cells were plated in 6-well plates (1 × 10^6^ cells per well) for 24 h and treated with various concentrations (10^−5^ to 10^−7^ M) of paeoniflorin or albiflorin for 18 h. After discarding the cell culture medium, cells were washed three times with precold PBS and lysed with a Nuclear and Cytoplasmic Protein Extraction Kit (Beyotime Institute of Biotechnology, China). For Western blot analysis, equal quantities (20 *μ*g) of total protein were subjected to SDS-PAGE and boiled for 5 min. Subsequently, samples were electrophoretically transferred to polyvinylidene difluoride (PVDF) membranes, blocked with TTBS (0.5% Tween 20, 10 mM Tris-HCl, pH 7.5, and 150 mM NaCl) containing 5% nonfat milk for 1 h at room temperature, and incubated with antibodies against GAPDH, CYP3A4, and CYP2D6 overnight at 4°C. Membranes were washed and further incubated with HRP-conjugated secondary antibodies against rabbit for 1 h at room temperature. After washing, protein bands were detected by Immobilon Western chemiluminescent HRP Substrate (Millipore, USA) and exposure to X-ray films. Films were scanned into a computer and densitometry of the image was quantified using an Image-Pro Plus Software Version 6.0 (Media Cybernetics, Silver Spring, MD, USA).

### 2.7. P450-Glo Assays

CYP enzyme activities were detected using P450-Glo CYP450 Screening Systems (Promega, Madison, WI, USA) according to the manufacturer's protocol.

### 2.8. Statistical Analysis

Statistical analyses were performed with Origin 7.5 software (Microcal Software, Inc., Northampton, MA, USA). For all experiments, data were expressed as means ± SD from three independent experiments. Differences between the values of various experimental groups were assessed with one-way analysis of variance (ANOVA) and a *P* value less than 0.05 was considered to be statistically significant.

## 3. Results

### 3.1. Effect of Paeoniflorin and Albiflorin on HepG2 Cells Viability

**Figure 2:**
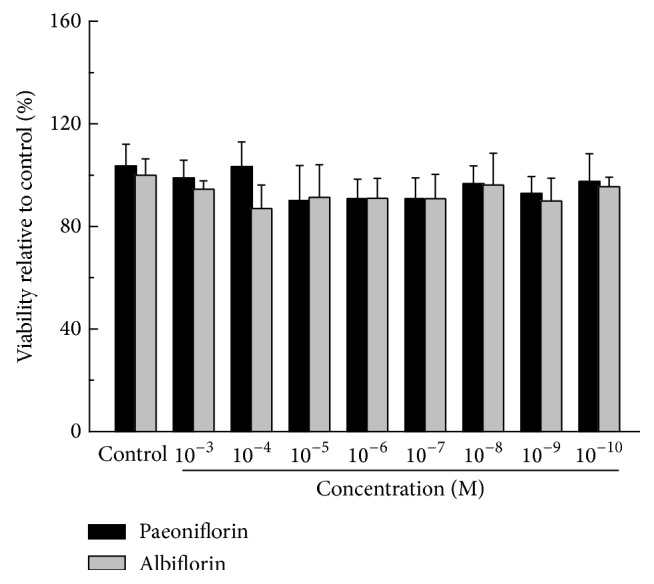
Cytotoxicity of paeoniflorin and albiflorin on HepG2 cells. Cells cultured in the 96-well plate for 24 h were incubated with paeoniflorin and albiflorin (10^−3^–10^−10^ M), respectively, for 18 h. After MTT reagent (10 *μ*L per well) was added for additional 2 h, the absorbance was recorded at 570 nm and cells viability was represented as the percent decrease compared to the control cells. Values are means ± SD (*n* = 6) from three independent experiments and there is no significant difference compared with control group cells.

Cytotoxicity of paeoniflorin and albiflorin on HepG2 cells was detected with MTT assay. Results showed that the cell viability was not significantly modified, no matter whether treated with paeoniflorin or albiflorin, compared with blank control group (Figure 2, *P* > 0.05). After oral gavage of peony to rats, the maximum plasma concentration of paeoniflorin and albiflorin was about 1248 *μ*g/L (equivalent to 2.6 × 10^−6^ M) and 1550 *μ*g/L (equivalent to 3.2 × 10^−6^ M), respectively [22]. However, the maximum plasma concentration of intravenous drug delivery may be 10-fold higher than that of oral gavage. According to the absorption of paeoniflorin and albiflorin* in vivo*, concentrations of 10^−5^ M to 10^−7^ M of paeoniflorin and albiflorin were selected for further study.

### 3.2. Effect of Paeoniflorin and Albiflorin on CYP3A4 and CYP2D6 mRNA Expression

**Figure 3:**
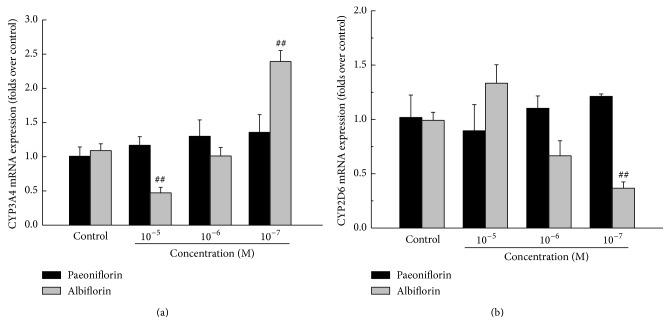
Effects of paeoniflorin and albiflorin on CYP3A4 and CYP2D6 mRNA expression. HepG2 cells, treated with paeoniflorin and albiflorin (10^−5^–10^−7^ M), were used to detect the variations of mRNA expressions of CYP isoenzymes. The mRNA expressions of CYP3A4 (a) and CYP2D6 (b) were analyzed by real-time RT-PCR. Values are means ± SD (*n* = 3) and significant difference compared with control group; ^##^ *P* < 0.01, albiflorin-treated groups versus control group.

As shown in Figure 3, paeoniflorin had no significant effect on mRNA expression of CYP3A4 and CYP2D6. However, albiflorin showed a wide regulation on them. In detail, high concentration of albiflorin (10^−5^ M) could markedly suppress the mRNA expression of CYP3A4 (43.4%, *P* < 0.01). But with the loss of the drug concentration, low concentration of albiflorin (10^−7^ M) aberrantly induced the mRNA expression of CYP3A4 by 219.6% (*P* < 0.01). For the assay of CYP2D6, the low concentration of albiflorin exerted the decreasing effect (37.1% in 10^−7^ M, *P* < 0.01).

### 3.3. Effect of Paeoniflorin and Albiflorin on CYP3A4 and CYP2D6 Protein Expression

**Figure 4:**
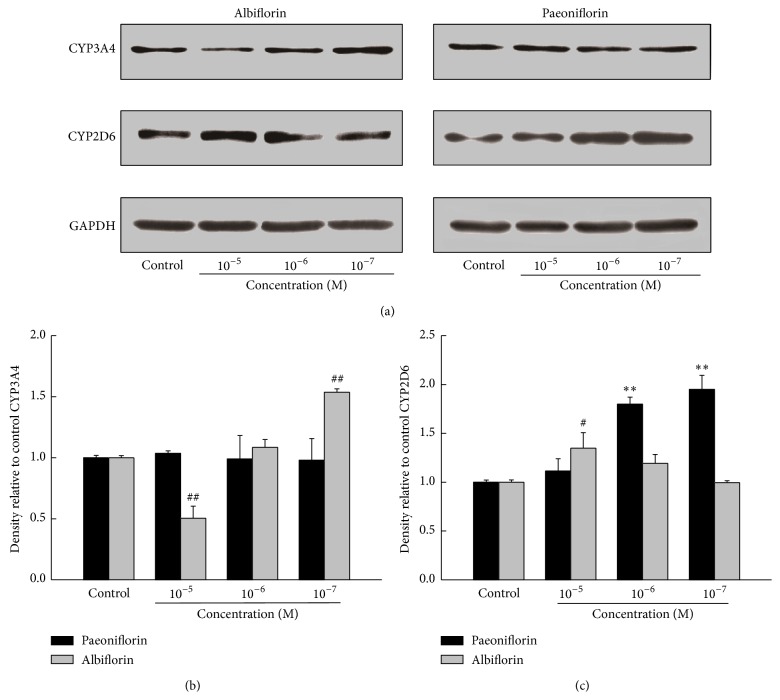
Effects of paeoniflorin and albiflorin on CYP3A4 and CYP2D6 protein expression. Protein expression of CYP3A4 and CYP2D6 in HepG2 cells was performed by Western blotting (a). The GAPDH was used as an internal control. Densitometry of the images of CYP3A4 (b) and CYP2D6 (c) was performed using the Image-Pro Plus Version 6.0. Values are means ± SD (*n* = 3) and significant difference compared with control group; ^#^ *P* < 0.05, ^##^ *P* < 0.01, albiflorin-treated groups versus control group; ^*∗∗*^ *P* < 0.01, paeoniflorin-treated groups versus control group.

Western blot analyses of CYP3A4 and CYP2D6 were conducted (Figure 4). Cells treated with albiflorin showed that the protein expression of CYP3A4 was decreased by high concentration of albiflorin (10^−5^ M, *P* < 0.01) and increased by a lower one (10^−7^ M, *P* < 0.01). However, paeoniflorin possessed no significant alteration on CYP3A4. Moreover, results indicated that both paeoniflorin (10^−6^ M and 10^−7^ M, *P* < 0.01) and albiflorin (10^−5^ M, *P* < 0.05) could significantly increase protein expression of CYP2D6. The GAPDH protein was used as an internal control.

### 3.4. Effect of Paeoniflorin and Albiflorin on CYP3A4 and CYP2D6 Enzyme Activities

**Figure 5:**
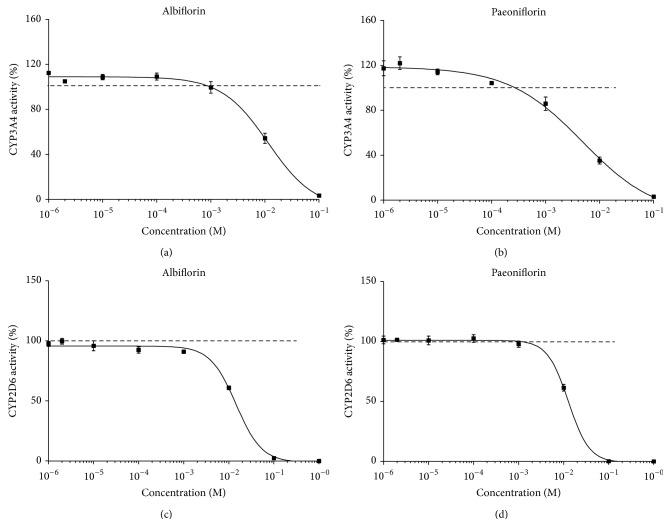
Effects of paeoniflorin and albiflorin on CYP3A4 and CYP2D6 enzyme activities. Enzyme activities of CYP3A4 and CYP2D6 were detected by P450-Glo Assays. Nonlinear regression analysis was performed to estimate the half-maximal inhibitory concentrations (IC_50_) and dashed lines represented 100% activity.

As shown in Figure 5, the half-maximal inhibitory concentration (IC_50_) of paeoniflorin was 4793 *μ*M and 12701 *μ*M for CYP3A4 and CYP2D6, and, in terms of albiflorin, they were replaced in turn by 11433 *μ*M and 14447 *μ*M. Nonlinear regression analysis also indicated that paeoniflorin and albiflorin possessed biphasic effects on CYP3A4. In detail, paeoniflorin and albiflorin with lower concentrations could induce CYP3A4 activity; however, the regulation turned into inhibition with the concentration increase. The low concentration of paeoniflorin had no significance on CYP2D6 activity, and an inhibition was found with the concentration increase. Unlike paeoniflorin, each concentration of albiflorin exerted broad inhibitory effect.

## 4. Discussion

The adverse effects of drug interactions induced by CYP induction or inhibition have been attracted wide attention [24]. Researchers have reported that the high content of licorice in shaoyao-gancao decoction could reduce the bioavailability of paeoniflorin and albiflorin [23]. For this phenomenon, no specific mechanisms have been provided. It might be because some ingredients in traditional Chinese medicine compound were substrates, inhibitors, or inducers of CYP450 isoenzymes and further affected the pharmacokinetic of each other. Peony is one of the most important herbs in traditional Chinese medicine, and it is vital to explore its characteristics. Previously, most researchers focused on the study of the total glucosides of peony to investigate the pharmacological actions. However, from the references review, more and more researchers focused on the difference between paeoniflorin and albiflorin. Recently, a few researchers reported that paeoniflorin, but not albiflorin, is the most important ingredient for anti-inflammation and neuroprotection [25].

In this study, we evaluated the regulation of paeoniflorin and albiflorin on CYP3A4 and CYP2D6 from different perspectives, including mRNA expression, protein expression, and enzyme activity. Comparing with paeoniflorin, the results showed that albiflorin was a strong regulator of CYP3A4 and CYP2D6 in HepG2 cell line and it might be indispensable for evaluating the medication safety of peony. P450-Glo Assay is a method mainly used to investigate dose-dependent CYP inhibition by test compounds against recombinant CYP enzymes* in vitro*. In this study, it was used to evaluate the regulation of paeoniflorin and albiflorin on CYP3A4 and CYP2D6 from the aspect of enzyme activity, which showed that both paeoniflorin and albiflorin could regulate the activity of CYP3A4 and CYP2D6 with varying degrees and the regulating range of albiflorin was wider than that of paeoniflorin. CYP3A4, the largest portion of P450 protein in human, plays a vital role in the metabolism of plenty of pharmaceutical products. CYP2D6 is a metabolic enzyme involved in approximately 25% clinical antidepressants and diazepam drugs. For example, sertraline is the substrate drug of CYP2D6 which induces adverse effect of severe liver damage. When peony was coadministrated with sertraline, the dose range and the extract components should be controlled. The induction of metabolic enzymes contributed to about 30% of drug interactions, while the inhibition accounts for the other 70%. Then, to evaluate the regulation of herbal components on CYP isoenzymes is valuable for the prediction of drug-drug interaction.

## 5. Conclusions

What we found in the present study might be another important reference for attaching great importance to albiflorin, because it was a stronger regulator for CYP3A4 and CYP2D6 than paeoniflorin, and it should be used with caution because of the potential risk of complex drug-drug interactions when coadministrated with other drugs or herbs. Based on the existing results, we proposed that albiflorin may play a vital role on the overall efficacy of peony. The whole behavior of both paeoniflorin and albiflorin should be focused on to ensure the rationality and effectiveness of clinical application.

## Figures and Tables

**Table 1:** The real-time RT-PCR oligonucleotide primers.

Gene	Primer	Sequence (5′-3′)	PCR product (bp)
GAPDH	Forward	CAATGACCCCTTCATTGACC	106
NM_002046.4	Reverse	GACAAGCTTCCCGTTCTCAG	
CYP3A4	Forward	CAAGACCCCTTTGTGGAAAA	187
NM_017460.5	Reverse	CGAGGCGACTTTCTTTCATC	
CYP2D6	Forward	CAGAGATGGAGAAGGCCAAG	191
NM_000106.5	Reverse	CCCTATCACGTCGTCGATCT	
